# Student Characteristics Associated with Passing the Exam in Undergraduate Pharmacology Courses—a Cross-sectional Study in Six University Degree Programs

**DOI:** 10.1007/s40670-020-01026-8

**Published:** 2020-07-13

**Authors:** Thomas Carlsson, Michael Winder, Anna L. Eriksson, Susanna M. Wallerstedt

**Affiliations:** 1 Department of Pharmacology, Sahlgrenska Academy, University of Gothenburg, Box 431, SE-405 30 Gothenburg, Sweden; 2 Department of Internal Medicine, Sahlgrenska Academy, University of Gothenburg, Gothenburg, Sweden; 3 Department of Clinical Pharmacology, Sahlgrenska University Hospital, Gothenburg, Sweden; 4 HTA Centre, Sahlgrenska University Hospital, Gothenburg, Sweden

**Keywords:** Examination, Higher education, Pharmacology, Student attendance, Student performance

## Abstract

Adequate knowledge in pharmacology is crucial in many professions but a non-negligible proportion of students fail the exams and knowledge of underlying factors is largely lacking. This study was performed to evaluate to what extent various factors are related to student performance in pharmacology-related courses in higher education, linking administrative data to attendance at non-mandatory teaching sessions and questionnaire replies. A total of 596 students (median age: 22 years; 70% female) were included from eight courses which are part of either the medical, pharmacy, dentistry, nursing, or biomedical analyst degree programs at the Sahlgrenska Academy, Gothenburg, Sweden. In all, 380 (64%) students passed the regular program- and course-specific exam. Multivariate logistic regression analysis revealed that a high participation rate in non-mandatory teaching sessions, as well as a perceived great interest in pharmacology, was associated with students’ passing of the exam; adjusted odds ratio (95% confidence interval): 1.30 (1.19 to 1.42; per 10 percentage unit increase in attendance) and 3.38 (1.86 to 6.12), respectively. Working for wages during the course weeks and pre-university grades used in the program application were significant factors in subgroups of students, negatively and positively associated with the exam results, respectively. Age, having Swedish as a second language, and time spent studying were only associated with the exam result in the univariate analyses. To conclude, both students and teachers can contribute significantly to successful education within pharmacology, students by participating in the teaching sessions and teachers by encouraging students to find the subject interesting.

**Electronic supplementary material:**

The online version of this article (10.1007/s40670-020-01026-8) contains supplementary material, which is available to authorized users.

## Background

Adequate knowledge in pharmacology is crucial in many professions, for example, within pharmacy, medicine, and nursing. Higher education is the basis for gaining this knowledge, and a passed exam for a specific course certifies that a student has reached the intended learning outcomes. However, as academic teachers in pharmacology-related courses, we experience that a non-negligible number of students nowadays fail the exams. A recent study from our institution shows that student performance in the pharmacy master’s degree program has indeed declined, with a decreased proportion of students passing the pharmacology exam [1]. Declining application rates and low academic performances have been reported also from other pharmacy schools, where intervention strategies have been developed and employed [2]. For informed decisions on how to improve student performance, an increased understanding of factors of importance for passing the exam is essential. Such knowledge may constitute a basis for further discussions on both student- and teacher-related educational aspects.

In the literature so far, much attention has been paid to the development of new teaching models, for instance flipped classroom, team-based learning, and audio response systems [3–5]. Furthermore, student health status and stress levels have been studied [6–8]. However, less is known regarding other student-related aspects associated with academic performance. For example, we have observed a decline in student attendance in the lectures over the years. A recent study, taking age and sex into account, suggests that student attendance in non-mandatory teaching sessions is associated with passing the exam [9]. We hypothesized that other aspects may also contribute to student performance in pharmacology-related courses, for instance pre-university grades, apprehensions of the subject per se, and time spent studying and working for wages in parallel to the studies. To the best of our knowledge, the importance of these factors for passing the exam has not previously been quantified. Therefore, we performed this study to evaluate to what extent these factors are related to student performance within pharmacology-related courses, linking administrative data to attendance at non-mandatory teaching sessions and questionnaire replies.

## Methods

To investigate student characteristics associated with passing the exam in undergraduate pharmacology courses, we performed a cross-sectional study in eight courses in six degree programs at the Sahlgrenska Academy, Gothenburg, Sweden (Table 1). The degree programs included in the study were the medical (5.5 years), pharmacy (master: 5 years; bachelor: 3 years), dentistry (5 years), nursing (3 years), and biomedical analyst (3 years) programs.

**Table 1:** Pharmacology courses included in the study

Course	Degree program	Semester	ECTS credits	Non-mandatory sessions (*n*)	Data collection
Pharmacology	Biomedical analyst	2	7.5	27	2019 (Mar 27–Apr 29)
Pharmacology (part of the course pharmacology/anesthesiology, examined separately)	Dentistry	5	5.5 (out of 7)	20	2018–2019 (Nov 13–Jan 8)
Pharmacology (part of the course physiology, pharmacology, and biochemistry, not examined separately)	Medical	2–3	9 (out of 40.5)	21	2019 (Mar 4–May 16)
Clinical pharmacology (part of the course internal medicine I and II, not examined separately)	Medical	6	1.5 (out of 37.5)	4	2019 (Feb 25–Mar 3/Mar 11–15)
Pharmacology and the handling of medication	Nursing	2	9	15	2018–2019 (Dec 7–Jan 18)
Integrative biomedicine II: pharmacology, basic medical concepts, and pharmacotherapy	Pharmacy, bachelor degree	3	18	42	2018 (Sept 3–Nov 14)
Pharmacology, medical chemistry, and biology of disease 2	Pharmacy, master degree	5	12	20	2018 (Sept 3–Oct 24)
Pharmacotherapy	Pharmacy, master degree	7	7.5	21	2018 (Oct 31–Dec 4)

*ECTS* European credit transfer and accumulation system

Data were collected from one cohort of students for each course. We informed the students about the study orally during the mandatory introduction. In addition, we posted this information in writing on the course-specific online student platform. All students in the selected courses were invited to participate. To stimulate participation, a cinema voucher was randomly offered to every third student completing the study. In the present study, students registered at the course for the first time were eligible. Attendance in non-mandatory sessions was noted continuously during the courses by passing a list through the auditorium, headed with the title and the date of the lecture, with instructions to participating students to write their names. No recorded lectures were provided in any of the courses. Students writing their names at ≥ 1 occasion were included in the study, this active choice reflecting informed consent. For each student, we recorded the proportion of non-mandatory sessions attended.

To obtain information on the students’ apprehensions of pharmacology, the extent of their time spent studying and working for wages during the course, as well as if they had forgotten to write their name on the attendance list on any occasion, we distributed an electronic questionnaire to all participants after the completion of the course (Appendix). Apprehension questions were formulated as Likert statements, to which the students were asked to grade their agreement (from 1 = fully disagree to 5 = fully agree). For questions regarding time spent studying and working, reply alternatives were provided in categories. In this study, we dichotomized the student responses, studying either ≥ 30 or < 30 h per course week and either working for wages or not.

To retrieve information regarding the students taking the regular program- and course-specific exam or not, as well as their passing or failing this exam, we used the university administrative system. For medical students, where pharmacology and clinical pharmacology are not separately examined, those taking the exam with 60% correct answers to the pharmacology questions were categorized as passing the exam. We used the unique personal identity number to determine age and sex. This number was also used to extract (i) the pre-university grades used in the granted program application and (ii) potential grades in Swedish as a second language. This information was extracted from the Swedish university admission system (NyA-webben, Universitet- och högskolerådet, Stockholm, Sweden).

### Statistics

The statistical analyses were performed with SPSS (IBM SPSS Statistics for Windows, version 23.0, Armonk, NY). In dichotomized analyses, we categorized students as fully agreeing (5), or less (1–4), to the Likert statements regarding their perceived interest in pharmacology as well as the perceived importance of this subject for their future professional life. Thereby, the analysis differentiated between those who were fully positive about the subject and those who were not. To investigate the association between student characteristics and results of the exam, we performed logistic regression analyses resulting in crude and adjusted odds ratios for passing the exam (as opposed to failing or not taking it), with 95% confidence intervals (CI). We included the following covariates in the analyses: age (in four categories: ≤ 20, 21–25, 26–30, ≥ 31 years of age), sex (female versus male), pre-university grades used in the granted program application (continuous variable; minimum approximately 10, maximum 22.5), Swedish as a second language (≥ 1 grade versus none), participation rate in non-mandatory teaching sessions (in ten categories from 0 to 100%), perceived interest in pharmacology (dichotomized), perceived significance of pharmacology for future professional life (dichotomized), time spent studying per course week (≥ 30 h versus < 30 h), and work for wages during the course (yes versus no). To detect potential multicollinearity issues between these factors, we investigated bivariate correlations with Pearson’s correlation coefficient and used the tolerance statistics in the regression analysis. To investigate the robustness of the results, we performed a sensitivity analysis in which students who stated that they had forgotten to write their names on the attendance list at least once were excluded. To elucidate the importance of pre-university grades, we also performed the multivariate logistic analysis after exclusion of medical and dentistry students. The rationale for this exploratory analysis was that high pre-university grades are required to enter the medical and dentistry programs, making students in these programs homogenous regarding this variable and thereby reducing the potential to reveal an association. To explore any differences between the programs, univariate logistic regression was performed to obtain odds ratios for passing the exam according to the two most important factors found in the multivariate model.

## Results

In all, 596 out of 674 eligible students were included in the study (participation rate: 88%). An additional 16 students wrote their names on the attendance lists but were not eligible as they had previously been registered for the course in question. Characteristics of participating students are presented in Table 2. Briefly, the students had a median age of 22 years and 420 (70%) were female. The majority were medical or pharmacy students (*n* = 411, 69%).

**Table 2:** Characteristics of students

		Total, *n* = 596	Exam result	
			Passed, *n* = 380	Failed^1^, *n* = 216
Age, median (range)		22 (19–51)	22 (19–49)	23 (19–51)
Female sex, *n* (%)		420 (70)	262 (69)	158 (73)
Degree program, *n* (%)	Biomedical analyst	71 (12)	38 (10)	33 (15)
	Dentistry	35 (6)	27 (7)	8 (4)
	Medical	246 (41)	152 (40)^2^	94 (44)^2^
	Nursing	79 (13)	27 (7)	52 (24)
	Pharmacy, bachelor degree	46 (8)	38 (10)	8 (4)
	Pharmacy, master degree	119 (20)	98 (26)	21 (10)
Pre-university grades used in program application^3^, median (range)		19.41 (9.74–22.50)	19.67 (9.74–22.50)	19.00 (11.28–22.50)
≥ 1 grade in Swedish as a second language, *n* (%)		119 (20)	63 (16)	56 (26)
Participation rate in non-mandatory teaching sessions, median (range)		0.75 (0.04–1)	0.86 (0.04–1)	0.52 (0.04–1)
Apprehensions in questionnaire, *n* (%)^4^	Was very interested in pharmacology	174 (40)	139 (45)	35 (28)
	Considered pharmacology very important for future professional life	316 (73)	226 (74)	90 (71)
	Spent ≥ 30 h per course week studying	279 (64)	208 (68)	71 (56)
	Worked for wages during the course weeks	184 (42)	121 (40)	63 (50)

The data are presented for all students in total and in two subgroups defined by their exam result, as median (range) and counts (percentages). Percentages were calculated within each of these groups. ^1^Including those who failed and those who refrained from writing the exam; ^2^pharmacology part of the syllabus but not examined separately; those with 60% correct answers were categorized as passing the exam; ^3^10 is the minimum grade to pass a course in upper secondary school and 22.5 is the maximum grade that can be used in the degree program application; ^4^percentage of respondents (total: *n* = 433; passed exam: *n* = 306; failed exam: *n* = 127)

A total of 380 (64%) students passed the exam, whereas 216 (36%) did not (failed: *n* = 149, 25%; refrained from taking the exam: *n* = 67, 11%). According to the completed attendance lists, 275 (46%) students participated in ≥ 80% and 165 (28%) attended ≤ 50% of the non-mandatory teaching sessions. Furthermore, 52 (9%) attended < 20% of the non-mandatory teaching sessions. The pre-university grades, used in the granted program application, ranged from below the level required to pass a course in upper secondary school (i.e., 10.00) to the maximum level (i.e., 22.50). Among students with pre-university grades available (*n* = 548), 76 (14%) had > 22 and 42 (8%) had < 15. According to recorded grades, 119 (20%) had Swedish as a second language.

In all, 433 students responded to the questionnaire (response rate: 73%). The median agreement, regarding perceived interest in pharmacology, was 5 (interquartile range: 4–5; mean: 4.1). The corresponding figure regarding the perceived significance of this subject for future professional life was also 5 (interquartile range: 4–5; mean: 4.6). Regarding time dedicated to studies, 223 (52%) students spent 30–50 h per week, 154 (36%) spent < 30 h, and the remaining 56 (13%) spent > 50 h. A total of 184 (42%) students reported that they had worked for wages in parallel to their studies; 127 (29%) worked 1–8 h per week, 39 (9%) worked 9–16 h per week, and 18 (4%) worked ≥ 17 h per week. The remaining 249 (58%) students reported that they did not work for wages during the course.

Logistic regression, with all factors included in the model, revealed that an increased participation rate in non-mandatory teaching sessions was associated with passing the exam (Table 3). Using the figure obtained in this analysis, a student participating in 90% of non-mandatory lectures would have 2.9 times higher chance of passing the exam, as compared with a student participating in 50%. Bivariate correlations between the factors included in the model were weak or very weak; 23 of 28 correlation coefficients were < 0.1 and the remaining five ranged between 0.11 and 0.37. The tolerance levels ranged between 0.83 and 0.99 for the various factors, indicating that multicollinearity was not a problem. In univariate analyses, participation rate was positively associated with passing the exam in all programs (Table 4). Being very interested in pharmacology was also positively associated with passing the exam, with increased odds of 3.38 in the multivariate analysis (Table 3). In univariate analyses, within each program separately, the point estimate was above 1 irrespective of program, with wide confidence intervals including the line of unity (Table 4). The perceived interest in pharmacology was greatest among pharmacy students and lowest among medical students. Overall, the sensitivity analysis, in which students who had forgotten to write their name on the attendance list ≥ 1 times were excluded (*n* = 123), revealed similar results (Table 3).

**Table 3:** Associations between student characteristics and passing the exam

		OR (95% CI)	aOR (95% CI)	aOR (95% CI)^1^
Age^2^		*0.82 (0.67 to 0.99)*	0.79 (0.59 to 1.07)	0.97 (0.68 to 1.39)
Female sex (vs. male)		0.82 (0.56 to 1.18)	*0.51 (0.29 to 0.92)*	0.68 (0.35 to 1.33)
Pre-university grades used in program application (continuous variable)		*1.08 (1.01 to 1.15)*	1.07 (0.98 to 1.18)	1.10 (0.99 to 1.23)
≥ 1 grade in Swedish as a second language		*0.57 (0.38 to 0.85)*	0.69 (0.37 to 1.30)	0.86 (0.36 to 2.04)
Participation rate in non-mandatory teaching sessions^3^		*1.33 (1.25 to 1.41)*	*1.30 (1.19 to 1.42)*	*1.28 (1.15 to 1.42)*
Apprehensions	Was very interested in pharmacology	*2.29 (1.40 to 3.43)*	*3.38 (1.86 to 6.12)*	*2.82 (1.41 to 5.61)*
	Considered pharmacology very important for future professional life	1.16 (0.73 to 1.84)	0.70 (0.39 to 1.26)	0.53 (0.26 to 1.10)
	Spent ≥ 30 h per course week studying	*1.67 (1.09 to 2.56)*	1.09 (0.66 to 1.80)	0.74 (0.40 to 1.38)
	Worked for wages during the course weeks	0.66 (0.44 to 1.01)	0.65 (0.39 to 1.07)	*0.52 (0.29 to 0.95)*

The data are presented as crude and adjusted odds ratios (OR and aOR) for passing the exam according to student characteristics, including 95% confidence intervals (CI). In the adjusted models, all variables were included. Significant associations are in italics. ^1^Sensitivity analysis in which students who had forgotten to write their name on the attendance list ≥ 1 times were excluded (*n* = 123); ^2^four categories: ≤ 20, 21–25, 26–30, ≥ 31 years of age; ^3^ten categories: 0–0.09, 0.10–0.19, 0.20–0.29, 0.30–0.39, 0.40–0.49, 0.50–0.59, 0.60–0.69, 0.70–0.79, 0.80–0.89, 0.90–1

**Table 4:** Associations between participation rate and subject interest with passing the exam according to degree program

		Results	
		Median (range)/*n* (%)	OR (95% CI)
Biomedical analyst	Participation rate in non-mandatory teaching sessions	0.67 (0.04–1)	*1.51 (1.23 to 1.87)*
	Was very interested in pharmacology	15 (33)	1.91 (0.44 to 8.30)
Dentistry	Participation rate in non-mandatory teaching sessions	0.70 (0.10–1)	*1.51 (1.08 to 2.11)*
	Was very interested in pharmacology	10 (38)	2.29 (0.10 to 16.3)
Medicine	Participation rate in non-mandatory teaching sessions	0.81 (0.05–1)	*1.25 (1.14 to 1.36)*
	Was very interested in pharmacology	34 (20)	2.41 (0.93 to 6.24)
Nursing	Participation rate in non-mandatory teaching sessions	0.73 (0.07–1)	*1.48 (1.15 to 1.90)*
	Was very interested in pharmacology	19 (32)	1.67 (0.55 to 5.07)
Pharmacy, bachelor degree	Participation rate in non-mandatory teaching sessions	0.68 (0.05–1)	*1.47 (1.08 to 1.99)*
	Was very interested in pharmacology	28 (78)	2.00 (0.29 to 13.6)
Pharmacy, master degree	Participation rate in non-mandatory teaching sessions	0.81 (0.05–1)	*1.45 (1.21 to 1.75)*
	Was very interested in pharmacology	68 (70)	1.35 (0.45 to 4.09)

The data are presented as median (range), counts (percentage of respondents), and crude odds ratios (OR) with 95% confidence interval (CI) for passing the exam. Significant associations are in italics. The “Participation rate in non-mandatory teaching sessions” data were divided into ten categories as follows: 0–0.09, 0.10–0.19, 0.20–0.29, 0.30–0.39, 0.40–0.49, 0.50–0.59, 0.60–0.69, 0.70–0.79, 0.80–0.89, 0.90–1

In the univariate, but not the overall multivariate, analysis, pre-university grades were associated with passing the exam. When medical and dentistry students were excluded, there was a significant association also in the multivariate analysis, adjusted odds ratio: 1.25 (95% CI: 1.08 to 1.46), and attendance in non-mandatory sessions and interest in pharmacology were still associated with passing the exam (1.42 (1.23 to 1.64) and 3.91 (1.77 to 8.62), respectively).

## Discussion

In this study, we found that a high participation rate in non-mandatory teaching sessions, as well as a perceived great interest in pharmacology, was significantly associated with students’ passing of the exam in pharmacology-related courses in higher education. The results were similar for all degree programs including medicine, pharmacy, dentistry, nursing, and biomedical analyst. Furthermore, working for wages during the course weeks and pre-university grades used in the program application were important factors in subgroups of students, negatively and positively associated with the exam results, respectively. Conversely, age, having Swedish as a second language, and time spent studying were only associated with the exam result in the univariate analyses.

The finding that attendance in non-mandatory teaching sessions is closely related to passing the exam is consistent with previous findings among nursing students [9]. Our results reveal that this conspicuous association remains also after relevant factors, other than age and sex which were accounted for in the previous study, have been considered. In the multivariate analysis, including only students with full attendance data, neither age nor sex was significantly associated with passing the exam. Knowledge of the association between attendance and the chance of passing the exam may be valuable for students, enabling informed decisions regarding their participation in non-mandatory teaching sessions in pharmacology courses in higher education. In fact, we were surprised that three in ten students participated in 50%, or less, of the non-mandatory teaching sessions and that one in ten skipped 80% of these sessions. Indeed, the learner’s roles during different phases of learning have been outlined, suggesting active participation when faced with the fact that one’s own knowledge is incomplete and when solutions for this are elaborated on [10]. However, as the availability of recorded lectures and other online material increase, the importance of classroom attendance may decrease, at least in some subgroups of students [11].

As could be expected, perceived interest in pharmacology was important for passing the exam. Although it may not be surprising that the interest for this core subject was greatest among pharmacy students, we found it somewhat surprising that two to three in ten pharmacy students did not declare to be very interested in pharmacology. Our results challenge not only the students to find an interest in this key subject of their higher education but also the teachers to stimulate the students’ interest. In fact, a positive relationship has been shown between teachers’ subject-specific enthusiasm and student performance [12]. Furthermore, enthusiasm has been demonstrated as an important teacher quality [13], and a well-slept teacher has been shown preferable for students’ academic motivation and in-class satisfaction [14]. Conversely, teachers being perceived as bored may reduce students’ learning motivation [15].

Interestingly, the majority of the students were fully aware of the importance of pharmacology knowledge for their future professional life. However, among medical students, the interest in this subject was surprisingly low; only one in five medical students perceived a great interest in pharmacology. This finding suggests that there may be room for improvement when it comes to pharmacology teaching in medical school, in particular as concerns have been raised that the current education does not sufficiently prepare medical students for their professional prescribing responsibilities [16–19]. To manage complex treatment decision-making at the patient level, a combination of medical and pharmacological knowledge is required. To promote active learning, integrating physiology, pathophysiology, and pharmacology in preclinical teaching, the use of virtual patients has been suggested [20]. At a later stage in medical education, training and collegial discussions have been shown to be key success factor [21–23]. A potential explanation for the low interest in pharmacology among medical students, not directly related to the teaching sessions per se, may be the absence of a separate exam. If not examined separately, the level of knowledge achieved by the students may be affected [24]. Furthermore, the absence of a specific exam may convey the perception that pharmacology has an inferior role in the program.

In the multivariate analysis, time spent studying was not significantly associated with exam result. Nevertheless, the finding that one in three students spent less than 30 h a week studying illustrates that the experienced work load to pass the exam may vary largely between students. The wide range in pre-university grades, reflecting basic level of knowledge, may support this assumption. Actually, work avoidance has been shown to be a minor issue [25]. Although pre-university grades were not significantly associated with passing the exam in the whole set, a significant association was found when medical and dentistry students were excluded. These results indicate that grades may be a more important predictor of academic success in student cohorts with a lower pre-university knowledge level. When all other variables had been considered, a student with pre-university grades of 20 (out of a maximum score of 22.5) had three-fold greater odds of passing the exam compared with a student with 15. The fact that four in ten students worked for wages during the course may also contribute to student performance. In the sensitivity analysis, including students with full attendance data, those working in parallel performed half as well in the exam. Finally, language aspects may be an issue. In the present study, all students aimed for regulated professions where Swedish is the primary language of communication. Although this factor was not significantly associated with exam result in the multivariate analysis, the confidence interval being wide, the point estimate was constantly below 1. Furthermore, as many as one in five students had Swedish as a second language. In summary, our results support that teaching in higher education today is a challenge both regarding student performance and the diverse backgrounds of students which have to be mastered [1].

## Strengths and Limitations

The main strength of this study is that it contributes knowledge on factors associated with passing an exam in a pharmacology-related course in higher education, taking relevant factors into account. The high participation rate contributes to an acceptable external validity. However, among those not signing any attendance list, only 41% took the exam and 15% passed. For those participating, 89% took the exam and 64% passed. This discrepancy needs to be considered in the interpretation of the results. Another strength is the inclusion of several educational programs, gaining similar results when analyzed separately, making it reasonable to believe that the results are applicable to pharmacology teaching and learning in general. Furthermore, the results were shown to be robust in a sensitivity analysis. An important limitation of the study is the cross-sectional design, which allows conclusions regarding associations but not causality. Furthermore, the lack of a statistically significant association between, for instance, time spent studying and passing the exam, does not preclude such as association. Indeed, the confidence intervals concerning some factors were quite wide and would have required a larger number of students to accept/reject an association.

## Conclusion

This study provides evidence valuable for students as well as teachers in higher education. For academic success, i.e., for students to pass the exam, participation in non-mandatory teaching sessions should be encouraged. Furthermore, the importance of stimulating the students’ interest in the subject could be emphasized. The results illustrate challenges within higher education; student performance and diverse backgrounds of students need to be recognized and both students and teachers can contribute to passing rates.

## Electronic supplementary material

ESM 1 (DOCX 14 kb)

## Data Availability

The data analyzed in the current study are available from the corresponding author on reasonable request.
