# Supplementary material for: Student Characteristics Associated with Passing the Exam in Undergraduate Pharmacology Courses—a Cross-sectional Study in Six University Degree Programs
Source: Med Sci Educ. 2020 Jul 13;30(3):1137–44. doi: 10.1007/s40670-020-01026-8 (PMC8368336; doi:10.1007/s40670-020-01026-8)
Supplement: Supplementary file 1 — (DOCX 14 kb) [file 40670_2020_1026_MOESM1_ESM.docx]

**Appendix** Questions included in the web-based questionnaire distributed after the course

| **Question** | **Text** | **Reply alternatives** |
| --- | --- | --- |
| 1 | I am very interested in pharmacology. | Level of agreement, from 1 (fully disagree) to 5 (fully agree) |
| 2 | I consider the subject pharmacology of great importance for my future professional life | Level of agreement, from 1 (fully disagree) to 5 (totally fully) |
| 3 | During this course, I studied ______ hours per week | 1: <30 h/week; 2: 30-50 h/week; 3: >50 h/week^1^ |
| 4 | During this course, I have been working for wages ______ hours per week | 1: 0 h/week; 2: 1-8 h/week; 3: 9-16 h/week; 4: >17 h/week^2^ |
| 5 | Have you, at any non-mandatory session during the course, participated but forgotten to write your name on the attendance list? | 1: No; 2: Yes, once; 3: Yes, two to three times; 4: Yes, more than three times^2^ |

^1^In bivariate analyses, reply 2 and 3 were aggregated

^2^In bivariate analyses, reply 2, 3, and 4 were aggregated
